# Supplementary material for: Multi‐dataset identification of innovative feature genes and molecular mechanisms in keratoconus
Source: J Cell Mol Med. 2024 Sep 19;28(18):e70079. doi: 10.1111/jcmm.70079 (PMC11412914; doi:10.1111/jcmm.70079)
Supplement: Supplementary file 2 — Material S2. [file JCMM-28-e70079-s002.zip › 7.GSEA analysis/4.MYRF/KEGG_HEDGEHOG_SIGNALING_PATHWAY.html]

Details for gene set KEGG\_HEDGEHOG\_SIGNALING\_PATHWAY[GSEA]

|  || Dataset | MYRF.group.cls#h\_versus\_l |
| Phenotype | group.cls#h\_versus\_l |
| Upregulated in class | l |
| GeneSet | KEGG\_HEDGEHOG\_SIGNALING\_PATHWAY |
| Enrichment Score (ES) | -0.43755108 |
| Normalized Enrichment Score (NES) | -1.4190407 |
| Nominal p-value | 0.044354837 |
| FDR q-value | 0.23317069 |
| FWER p-Value | 0.861 |
Table: GSEA Results Summary

  

Fig 1: Enrichment plot: KEGG\_HEDGEHOG\_SIGNALING\_PATHWAY      
 Profile of the Running ES Score & Positions of GeneSet Members on the Rank Ordered List

  

| SYMBOL | TITLE | RANK IN GENE LIST | RANK METRIC SCORE | RUNNING ES | CORE ENRICHMENT || 1 | IHH | na | 1463 | 0.404 | -0.0015 | No |
| 2 | LRP2 | na | 2004 | 0.356 | 0.0189 | No |
| 3 | ZIC2 | na | 3808 | 0.261 | -0.0069 | No |
| 4 | CSNK1G1 | na | 4302 | 0.244 | 0.0036 | No |
| 5 | BMP8B | na | 4981 | 0.223 | 0.0065 | No |
| 6 | WNT10B | na | 5576 | 0.206 | 0.0101 | No |
| 7 | PTCH1 | na | 6108 | 0.189 | 0.0138 | No |
| 8 | WNT16 | na | 8449 | 0.128 | -0.0408 | No |
| 9 | CSNK1A1 | na | 8757 | 0.121 | -0.0375 | No |
| 10 | WNT5A | na | 9048 | 0.115 | -0.0342 | No |
| 11 | RAB23 | na | 9600 | 0.104 | -0.0397 | No |
| 12 | PRKX | na | 11663 | 0.068 | -0.0923 | No |
| 13 | SUFU | na | 11793 | 0.066 | -0.0893 | No |
| 14 | BMP8A | na | 12293 | 0.059 | -0.0977 | No |
| 15 | WNT3 | na | 12453 | 0.057 | -0.0966 | No |
| 16 | WNT2 | na | 13551 | 0.043 | -0.1238 | No |
| 17 | PTCH2 | na | 15175 | 0.026 | -0.1680 | No |
| 18 | HHIP | na | 15667 | 0.021 | -0.1800 | No |
| 19 | FBXW11 | na | 16395 | 0.014 | -0.1996 | No |
| 20 | BTRC | na | 16745 | 0.011 | -0.2086 | No |
| 21 | CSNK1G2 | na | 17793 | 0.001 | -0.2387 | No |
| 22 | WNT8A | na | 17911 | 0.000 | -0.2420 | No |
| 23 | GSK3B | na | 19466 | -0.016 | -0.2852 | No |
| 24 | PRKACA | na | 19584 | -0.017 | -0.2869 | No |
| 25 | CSNK1D | na | 20395 | -0.026 | -0.3076 | No |
| 26 | WNT4 | na | 20821 | -0.031 | -0.3167 | No |
| 27 | WNT3A | na | 20924 | -0.033 | -0.3164 | No |
| 28 | WNT9A | na | 21202 | -0.036 | -0.3208 | No |
| 29 | GLI3 | na | 21836 | -0.044 | -0.3346 | No |
| 30 | BMP7 | na | 22323 | -0.050 | -0.3436 | No |
| 31 | CSNK1A1L | na | 22742 | -0.056 | -0.3501 | No |
| 32 | CSNK1G3 | na | 23723 | -0.071 | -0.3711 | No |
| 33 | GAS1 | na | 23797 | -0.072 | -0.3660 | No |
| 34 | SMO | na | 25095 | -0.095 | -0.3938 | No |
| 35 | DHH | na | 26456 | -0.122 | -0.4207 | No |
| 36 | WNT1 | na | 27000 | -0.135 | -0.4227 | Yes |
| 37 | BMP6 | na | 27515 | -0.148 | -0.4226 | Yes |
| 38 | STK36 | na | 27892 | -0.157 | -0.4176 | Yes |
| 39 | GLI2 | na | 27939 | -0.159 | -0.4029 | Yes |
| 40 | WNT10A | na | 28144 | -0.164 | -0.3922 | Yes |
| 41 | WNT2B | na | 28930 | -0.189 | -0.3958 | Yes |
| 42 | WNT7B | na | 29404 | -0.203 | -0.3890 | Yes |
| 43 | BMP5 | na | 29906 | -0.220 | -0.3813 | Yes |
| 44 | WNT8B | na | 30486 | -0.242 | -0.3736 | Yes |
| 45 | PRKACG | na | 31385 | -0.285 | -0.3708 | Yes |
| 46 | WNT9B | na | 31651 | -0.298 | -0.3484 | Yes |
| 47 | WNT5B | na | 32169 | -0.328 | -0.3303 | Yes |
| 48 | WNT7A | na | 32275 | -0.335 | -0.2996 | Yes |
| 49 | GLI1 | na | 32884 | -0.379 | -0.2789 | Yes |
| 50 | WNT6 | na | 33111 | -0.398 | -0.2453 | Yes |
| 51 | PRKACB | na | 33438 | -0.435 | -0.2109 | Yes |
| 52 | BMP4 | na | 33633 | -0.460 | -0.1701 | Yes |
| 53 | SHH | na | 33799 | -0.486 | -0.1259 | Yes |
| 54 | WNT11 | na | 34512 | -0.683 | -0.0776 | Yes |
| 55 | BMP2 | na | 34656 | -0.831 | 0.0021 | Yes |
Table: GSEA details [plain text format]

  

Fig 2: KEGG\_HEDGEHOG\_SIGNALING\_PATHWAY      
 Blue-Pink O' Gram in the Space of the Analyzed GeneSet

  

Fig 3: KEGG\_HEDGEHOG\_SIGNALING\_PATHWAY: Random ES distribution      
 Gene set null distribution of ES for **KEGG\_HEDGEHOG\_SIGNALING\_PATHWAY**
